# Incidence, Impact, and Complications of Short Cephalomedullary Nail Toggling in Patients with Wide Femoral Medullary Canal

**DOI:** 10.3390/jcm14113961

**Published:** 2025-06-04

**Authors:** Ahmed Nageeb Mahmoud, Maria F. Echeverry-Martinez, Catherine Mary Doyle, Juan David Bernate, Michael Suk, Daniel Scott Horwitz

**Affiliations:** 1 Geisinger Medical Center, Danville, PA 17821, USA; 2 Faculty of Medicine, Ain Shams University, Cairo 11455, Egypt; 3 School of Medicine and Health Sciences, University of Rosario, Bogotá 111221, Colombia

**Keywords:** intertrochanteric femur fracture, short cephalomedullary nail, short PFN, trochanteric fracture, toggle

## Abstract

**Background:** Toggling of the short cephalomedullary nail is an understudied phenomenon characterized by a change in the longitudinal axis of the nail in relation to the longitudinal axis of the femoral medullary canal, with subsequent potential loss of reduction. This retrospective study aims to examine the incidence and impact of toggling of short cephalomedullary nails in cases with wide femoral canals. **Methods:** One thousand two hundred fifty-six (1256) cases that received short proximal femoral nails for intertrochanteric fractures were reviewed. Of them, 101 cases that had wide femoral canals (≥15 mm) and a minimum radiographic follow-up of 6 weeks were included in this study. Outcome measures included nail toggling, varus malunion and revision surgery. **Results:** After a mean radiographic follow-up of 53.5 weeks, sixteen cases (15.8%) showed significant nail toggling of more than 4 degrees and had subsequent varus displacement of the fracture. In all 16 cases, there was deficient proximal nail fixation, in the form of either a lag device not engaging the lateral wall (2 cases), lateral proximal femoral wall fracture/incompetency (7 cases), or a combination of the two factors (7 cases). Despite this, all sixteen cases achieved fracture union. Five additional cases had complications related to poor initial reduction (four cases) or femoral head avascular necrosis (one case). The other 80 cases had minimal (0–4 degrees) nail toggling and healed without varus malunion, and none of them required revision surgery. **Conclusions:** Short cephalomedullary nails may toggle in patients with wide femoral canals. The effect of femoral canal width on nail movement and subsequent varus malunion may be abolished when the lag device engages the lateral proximal femoral cortex, and the lateral cortical bone is intact. In patients with wide femoral medullary canals or cases with proximal lateral femoral cortical fracture, the utilization of long or intermediate length cephalomedullary nails may be a more viable option.

## 1. Introduction

In the management of intertrochanteric fractures (AO/OTA 31, A1–A3) [1], intramedullary devices such as cephalomedullary nails (CMNs) have increasingly become the preferred implants by many surgeons due to their relatively easier surgical technique, smaller incisions and biomechanical advantages compared to sliding hip screws, especially in patients with unstable fracture patterns [2,3,4,5,6,7,8].

Whether to use short or long CMNs in intertrochanteric fractures remains debatable and is influenced by many factors, including fracture pattern, bone quality, and surgeon preference [2]. As there are no significant differences in outcomes in the literature between the two implant types [9,10,11,12], short nails (SNs) are widely used owing to the shorter surgery duration, relative ease of distal interlocking, lower blood loss, and potential decreased cost [12,13,14]. Conversely, many surgeons typically prefer long nails in low intertrochanteric fractures that extend distal to the lesser trochanter (AO/OTA 33 B2.2 and B2.3, and B3), when the fracture configuration shows that short nails may not provide adequate fixation [15]. Challenges associated with short nail use are the limited utility and rotational stability in low and unstable trochanteric fractures, and the increased mid-femoral stresses at the distal end of the nail. Long nails also possess several challenges, including longer surgical times and blood loss, potentially increased costs, the risk of anterior femoral perforation in curved femurs, and the relatively more difficult revisions [2,3,7,8,9,10,11,12,13,14,15,16].

In patients with relatively wide proximal femoral medullary canals, the potential for toggling of the distal end of a short CMN is an increasing concern [3,16,17]. Nail toggling is defined as the movement of the nail around the distal interlocking screw with subsequent potential loss of reduction [17]. In a broader definition, it can be defined as a change in the longitudinal axis of the nail in relation to the long axis of the femoral shaft.

In a recent study of short nail toggling by George et al. [17], varus collapse, presented as the varus change in the femoral neck shaft angle from the intraoperative AP fluoroscopy to the final follow-up AP radiograph, was used as a surrogate for loss of reduction. According to their study, significant nail toggling (as defined as nail movement of more than 4 degrees) was associated with greater varus collapse of the fracture (6.2 degrees vs. 1.3 degrees) when compared to the patients who did not encounter significant nail toggling. The authors also found that the patients who had smaller nail diameter/medullary canal diameter ratio (0.54 vs. 0.74), and deficient proximal fixation in the form of smaller tip apex distance (TAD 13.4 vs. 18.5 mm), and/or lag device not engaging past the lateral proximal femoral cortex, had significant nail toggling. Since these results largely align with our experience, this retrospective study aims to examine the incidence, amount, and significance of short nail toggling in intertrochanteric fractures (AO/OTA 31, A1–A3) in a larger case series of patients with capacious medullary canals.

## 2. Materials and Methods

### 2.1. Study Design

After institutional review board (IRB) approval, the electronic medical record of our health system was reviewed using the surgical codes of proximal femoral nailing and the diagnostic codes of proximal femur fractures. The available patients’ encounters were individually evaluated for available radiographs and clinical data. After the exclusion of duplicate encounters, a total of 1256 cases that have received short proximal femoral nailing procedures were reviewed for this study. The radiographic and clinical data were examined against inclusion and exclusion criteria.

### 2.2. Inclusion Criteria

1. Intertrochanteric fractures (AO/OTA 31, A1–A3) treated with a Short CMN, and
2. Cases with a wide medullary canal that could potentially allow for nail motion/toggling and allow for easier detection/calculation of the toggling. Due to the absence of a clear definition of a wide medullary canal, and considering the risks of nail toggling in the study of George et al. [17], a medullary canal was considered wide in cases of:
  - Any Dorr [18] C case
  - Dorr B case with a medullary canal width of 15 mm or more.

Given that the available nail diameters are typically 9–12 mm, and to easily calculate the nail toggling, we chose a minimum canal width of 15 mm to allow >2 mm room for nail toggling in case the largest available nail was utilized. The medullary canal width is typically measured at 10 cm distal to the level of the mid-lesser trochanter after correction of magnification, based on the known nail diameter.

The decision to use a short nail was based primarily on the surgeon’s choice and the fracture configuration. In our institution, short CMNs have been utilized for intertrochanteric fractures when the fracture line is proximal or extended within 1–2 cm distal to the lesser trochanter.

### 2.3. Exclusion Criteria

- Patients with missing immediate post-operative or follow-up X-rays.
- Patients who did not complete a minimum radiographic follow-up of 6 weeks.

The Dorr Index, represented by the canal to calcar (CC) ratio [18] and medullary canal width [17], were primarily measured for all patients on the immediate postoperative AP radiographs in order to identify the included cases. Since the Dorr classification was originally described for use in arthroplasty cases with intact femurs [18,19], we have performed the CC ratio measurements on the first postoperative radiograph after fixation to avoid the effect of rotation in unreduced fracture cases.

A total of 1155 cases have been excluded due to being either Dorr A or Dorr B cases with narrow canal width, leaving 101 cases (96 patients, 5 bilateral) with wide femoral medullary canals and complete follow-up data for radiographic analysis. Follow-up was calculated according to the date of the last available plain radiographs that allowed for radiographic measurements. Performing the last measurements on radiographs that were taken at a similar postoperative period in all patients (at 12 weeks, for example) would have been an ideal method for measurement. However, since not all the patients had their radiographs taken at the same durations, and to follow the nail toggling until the last recorded follow-up, utilizing the last available radiographs was the most suitable option for this retrospective study.

The following data were obtained for each case: date of birth, date of surgery, age at time of surgery, date of the last available relevant radiographs, sex, and side. For each set of radiographs (the immediate postoperative and last available AP and lateral hip and femur radiographs)**,** the following measurements have been performed by three authors, among them two orthopedic surgeons, using Philips^®^ Intellispace Radiology Enterprise-4 software (Koninklijke Philips N.V., Amsterdam, The Netherlands):

- Tip apex distance (from the tip of the lag device to the femoral head apex), calculated from both AP and lateral radiographs.
- Nail/femoral canal angle. Cases with significant toggling, as defined by George et al. [17] as a change in the nail/femur angle of more than 4 degrees, were documented.
- Distance between the medial tip of the distal end of the nail and the endosteal border of the lateral femoral cortex.
- Varus displacement/malunion was identified as a change in the fracture alignment with a varus change in the neck shaft angle of more than 5 degrees [17].

Other measurements [17] made on the immediate postoperative radiograph included.

- Medullary canal width: Calculated at 10 cm from the tip of the lesser trochanter or tangent from the other side’s lesser trochanter tip, parallel to the inter-teardrop line.
- Nail Diameter.
- Nail/canal ratio.
- Engagement of the lateral aspect of the lag device past the lateral cortex. A lag device was identified as “engaged” when both the superolateral and inferolateral borders of the device protruded freely outside the lateral femoral cortex in the intraoperative radiographs. This measurement was particularly made in the intraoperative radiograph to document the status of lag device engagement before any weight bearing and potential fracture collapse, to judge the utilized surgical technique.
- Integrity of the proximal femoral lateral cortex: The proximal femoral lateral wall, where the lateral end of the lag device engages, was assessed in the preoperative, intraoperative, and immediate postoperative X-rays or CT scan. Lateral wall incompetency was considered if any lateral wall breakage or fissure fracture was noticed.
- Quality of reduction [17,20,21]: Good reductions were defined as having <4 mm of fragment displacement, a neck-shaft angle of < 5 degrees of varus or <20 degrees of valgus, and <20 degrees angulation on the lateral view radiographs. Acceptable reductions met the criteria for either alignment or displacement. Poor reductions met neither of the criteria [17]. Assessment of the reduction quality was retrospectively based on the last available C-arm radiographs.

Any detectable radiographic complications, including varus malunion (varus displacement of the neck shaft angle at the last follow-up radiographs), hardware cut out or migration (lag device cutting through, or sliding through the femoral head), loss of fixation (hardware failure), or revisions were recorded. The primary goal of this study is to review the radiographic outcomes related to nail toggling. Assessment of the functional outcomes was not the focus of this study.

### 2.4. Statistical Analysis and Data Interpretation

Data analysis was performed by Microsoft Excel (Microsoft Corporation, Redmond, WA, USA). Qualitative data were described using numbers and percentages. Quantitative data were described using mean and SD, median, and range. The Kolmogorov–Smirnov test was used to test data distribution normality. The Wilcoxon signed-rank, Unpaired *T*-test, Mann-Whitney U, and Fisher’s Exact tests were utilized, as appropriate, to compare the measurements and proportions between the groups. Statistical significance of the obtained results was judged at the (≤0.05) level.

## 3. Results

### 3.1. Demographics, Measurements, and Overall Outcomes

One hundred and one cases (96 patients, 67 females) fit the selection criteria. The mean age of the cases at the time of surgery was 83.2 years (range, 53–97.5), and 51 cases had the left side involved. All the patients had intertrochanteric fractures (AO/OTA 31-A1-A3), and none of the cases had inappropriately indicated short CMNs. All cases received short CMN (the majority received a size 11 nail, ranging from 9 to 12), and all of the implants were allowed to dynamically slide, as per manufacturers’ recommendations. The mean medullary canal diameter in all cases was 18.1 (range, 15.1–28). The mean nail/canal ratio for all cases was 0.6 (range 0.39–0.75). The mean follow-up until the last available relevant radiographs was 53.5 weeks. Twenty-two cases (21.7%) had a lag device not engaging the lateral femoral cortex, eight cases (7.9%) had a fractured/incompetent proximal femoral lateral cortex, and another eight cases (7.9%) had a combination of the two factors. The quality of reduction was graded as good in 63 cases, acceptable in 34, and poor in 4 cases. The mean immediate postoperative TAD for all cases was 15.9 (range, 4.8–24.7).

A total of 21 cases had complications related to either nail toggling (16 cases), poor reduction (4 cases), or femoral head avascular necrosis (AVN) (1 case). For all the other 80 cases that did not encounter complications, the mean nail toggle measurements (Nail/shaft angle and nail tip-femoral cortex distance) between the immediate postoperative and last available radiographs have slightly increased, which was found to be statistically significant (Table 1). This indicates an overall slight nail movement/toggling inside the femoral canal, which usually stops upon complete fracture healing or until the distal end of the nail impinges the femoral cortex laterally. For the tip apex distance, changes in the values were also statistically significant (Table 1), indicating a potential effect of nail toggling on the lag device tip apex distance. Neither the nail/canal ratio nor the immediate postoperative tip apex distance significantly differed in cases that had toggling more than 4 degrees or the non-complicated cases (Table 2).

### 3.2. Complications

**Figure 1:**
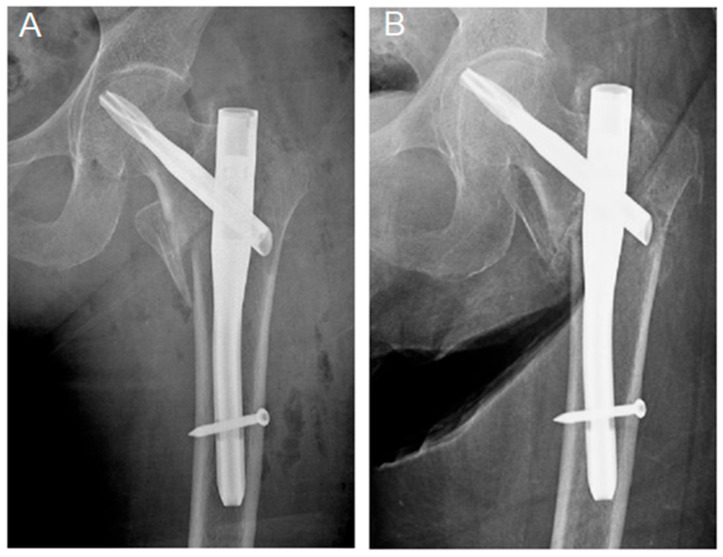
(**A**) Immediate post. (**B**) 3-month postoperative radiographs of an 81-year-old female with intertrochanteric fractures with improper proximal fixation (lag device not engaging an incompetent lateral femoral cortex) of a short cephalomedullary nail. The last radiograph (**B**) shows varus fracture displacement and lag screw articular penetration. The patient declined further interventions and expired 5 months later due to heart disease.

**Figure 2:**
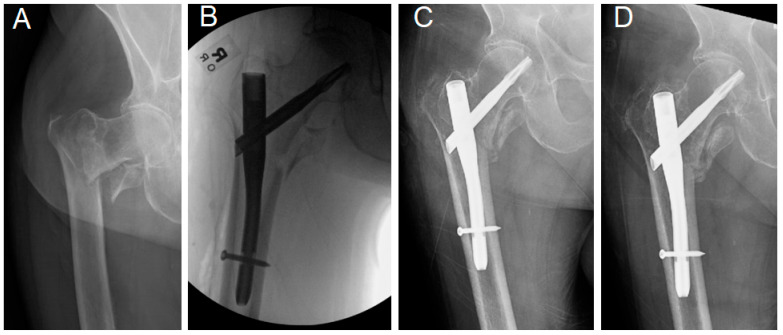
(**A**) Preoperative, (**B**) Immediate postoperative plain radiographs of 1 95-year-old male with an intertrochanteric fracture. Note the fractured/incompetent lateral wall. Note also that the lag device does not perfectly engage the lateral wall. (**C**) 8-week and (**D**) 16-week follow-up radiographs showing nail toggling and varus fracture collapse.

Significant nail toggling of more than 4 degrees with subsequent varus fracture collapse was observed in sixteen cases (Figure 1 and Figure 2). All 16 cases had a defect in proximal nail fixation, in the form of either a lag device not engaging the lateral wall (2 cases), lateral proximal femoral wall fracture/incompetency (7 cases), or a combination of the two factors (7 cases) (Table 3 and Table 4). Eventually, all sixteen cases achieved fracture union (varus malunion), which was minimally symptomatic given the patients’ age and activity levels. There were five other complications in the form of femoral head AVN and revision THA at 15 months in one case, and varus malunion in another four cases that had poor initial reduction.

**Figure 3:**
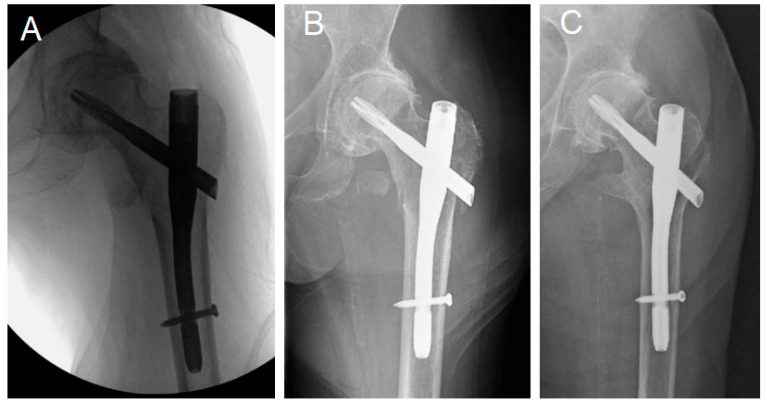
(**A**,**B**) Intraoperative and Immediate post. (**C**) 71-week postoperative radiographs of a 91.8-year-old female with a wide medullary canal and uneventful fracture healing without nail toggling despite a proximal lateral wall fracture.

The other 80 cases healed and had minimal nail toggling (Figure 3). None of them showed varus malunion, hardware failure, infections, hardware migration, or required revision surgery.

## 4. Discussion

This study aimed to evaluate the incidence and clinical implications of nail toggling in a series of 101 cases involving short proximal femoral nailing for intertrochanteric fractures in patients with wide femoral medullary canals. Out of 96 cases that had good or acceptable initial reduction and didn’t have AVN, 16 cases (16.7%) showed significant nail toggling and varus displacement, and all of them had deficient proximal nail fixation. There were five additional complications in patients who had poor initial reduction or AVN. All remaining cases healed without varus fracture displacement.

Short proximal femoral nail toggling has not been described frequently in the literature. The phenomenon has been discussed in two review articles [2,16] and one clinical study [17]. In the clinical retrospective study of 71 cases that received short proximal femoral nails, George et al. [17] found significant nail toggling with nail varus angulation of more than 4 degrees in 7 cases (9.8%). However, the majority of cases in the George et al. [17] study were Dorr B cases (57 cases, 80%), in whom large variations in the medullary canal size exist. In our study, we tried to further confine the study to include only cases with unequivocally wide femoral canals to adequately assess the occurrence and effect of toggling. To achieve that, we have included Dorr C cases, and Dorr B cases with a minimal canal width of 15 mm, to include all relevant cases and include any case that potentially allows room for nail movement in cases with larger nail diameters, reflecting the results of George et al., who identified a nail/canal diameter of less than 0.54 as a risk factor for significant nail toggling, and considering the most available nail sizes (9–12 mm). In the current study, significant nail varus toggling of more than 4 degrees with fracture varus displacement was observed in 16 cases, and all of them had lateral unsupported lag devices proximally (Table 3). This supports the finding in the previous study [17], which identified lag screw not engaging the lateral cortex as a risk factor for varus toggling of the nail. In cases where incompetent proximal fixation is suspected either pre- or intraoperatively, the use of a long CMN seems to be a more reasonable option. If the integrity of the proximal lateral femoral wall, where the lag device is contained, is questionable in the preoperative plain radiographs, it may be advisable to perform a CT scan to better understand the fracture geometry. It may also be advisable that whenever a short CMN is used, a lag device with an appropriate length that extends at least 2 mm past the lateral femoral cortex is used. This should allow for adequate fracture compression without the lag device entrapment or perching by the femoral cortex, which potentially blocks the collapse of the device.

Several factors could be identified as risk factors for increased short nail toggling and subsequent varus collapse. In the study of George et al. [17], the authors identified shorter tip apex distance, lag screw not engaging the lateral femoral cortex, Dorr C cases (wide medullary canals), and small nail/canal ratio as risk factors for significant nail toggling and subsequent varus malunion. The authors also discussed the shape of the distal locking screw hole as another potential risk factor for nail toggling, since oblong holes do not provide tight circumferential fit around the screw even when placed in static mode, allowing coronal plane toggling. To note, the absence distal locking screw is a potential risk factor for short nail toggling, which has been proven in two biomechanical studies [22,23].

Given that the toggling phenomenon has been reported exclusively with short nails, nail length seems to be another potential risk factor for toggling. With longer nails (long or intermediate length nails), the presence of additional medullary fit would theoretically prevent significant toggling [24], unless complete failure of the femoral shaft occurs. In addition, our study also suggests that the presence of proximal lateral wall fracture/incompetency is a strong risk factor for short nail toggling in wide medullary canals. Given the high incidence of complications associated with these risk factors (Table 3 and Table 4), whether they exist separately or in combination, a proper surgical technique should be followed to minimize the possibility of significant nail toggling and subsequent varus malunion.

Regarding the tip apex distance (TAD), despite a shorter distance indicating potentially better fixation into the femoral head, a smaller TAD may increase the lever arm on the nail compared to a lag screw with larger tip apex distance and may explain the findings of the previous study [17]. We would propose that a proper TAD (<25 mm) with lateral wall compression device engagement with an intact lateral cortex is the ideal situation.

In our results, the main factor determining the incidence and amount of toggling is the integrity of proximal nail fixation, rather than the medullary cavity size (Table 2). Based on our findings, nail toggling or lateralization of the distal portion of the nail does occur in a small amount, as the room allowed for nail movement is relatively small. This would result in only a few degrees of toggling that stops with fracture healing or when the distal end of the nail adjoins the femoral medullary wall. This assumes that the surgeon utilized a sound surgical technique, in the form of preoperative planning based on the bone and fracture configuration (lateral wall integrity), achievement of an acceptable near anatomic reduction, utilization of a nail with adequate size, utilization of a lag device with adequate length, adequate proximal fixation in the form of a tip apex distance less than 25 mm and a lag device engaging the lateral cortex, and utilization of locking screws of appropriate length. This also agrees with the study of Abram et al. [25], who found that inadequate proximal 3-point fixation in gamma nails was associated with increased incidence of fixation failure, nail fracture, or nail subsidence. Interestingly, the authors found that the strongest predictor of failure is lag device non-engaging the lateral cortex [25]. To note, in all of our cases, significant nail toggling was observed within the first 6 weeks postoperatively, and hence, a 6-week window may be sufficient to notice implant toggling, unless a recent significant trauma may occur.

Toggling, despite being recognized primarily in the coronal plane, can occur in the sagittal plane as well. In a biomechanical study by Kane et al. [26], the authors found that a cephalomedullary nail without distal locking screw is significantly more amenable to nail toggling in the coronal and sagittal planes. In another biomechanical study by Usami et al. [27], the authors investigated the effect of proximal femoral posterolateral fracture line morphology on intramedullary nail stability in unstable trochanteric fractures. The authors found that a proximal femoral fracture line that interferes with the lag screw insertion holes is a risk factor for increased intramedullary nail instability in the coronal and sagittal planes. In the sagittal plane, this leads to loss of cortical contact and support at the anteromedial inferior corner of the proximal femoral fragment, with subsequent varus collapse [27]. Based on the previous work of George et al. [17], our study focused only on the coronal plane toggling. A more comprehensive study should ideally evaluate toggling in both the coronal and sagittal planes.

Given that multiple studies have found short nails to be as effective as long nails, and with the longer operative time, potential higher cost, and increased blood loss associated with long nails [9,10,11,12,13,14,15], the results of this study support the use of short nails in patients with intertrochanteric fractures and wide femoral canals, whenever competent proximal fixation, suitable nail size, and proper surgical technique are achieved. In cases where proximal fixation is questionable in the setting of proximal lateral femoral wall incompetency, there is a significant risk of nail toggling and subsequent varus malunion with short CMNs in patients with a wide femoral canal. In such cases, the utilization of intermediate length [28,29,30,31,32,33] or long [34,35,36,37,38] nails could be a more reasonable option than choosing short nails, especially in cases with fracture extension more than 3 cm distal to the lesser trochanter [39,40]. Other operative factors to prevent nail toggling include the use of a properly engaged lag device with sufficient TAD, utilization of a distal locking screw, utilization of a well-fitting nail relative to the size of medullary cavity [16,17] and ensuring proper fracture reduction [41].

This study has several limitations, being retrospective and not comparative. The number of cases also remains relatively small, considering the fracture and implant popularity. Larger multicenter studies comparing the outcomes of short versus long nails in patients with wide femoral canals would potentially elicit more evidence. Another limitation is that we did not analyze the magnitude or outcomes of toggling in the sagittal plane, as we believed that coronal plane toggling is a representation of the overall toggling, which may not be the case [26,27,42,43]. Ideally, a study that analyzes both coronal and sagittal plane toggling would help to further understand the toggling phenomenon and identify the possible ways to avoid it. Finally, standardization of the radiographs and performing the measurements on true AP hip radiographs is ideal for proper comparisons and calculations of the amount of nail toggling. Given the retrospective nature of our study, we have tried to perform the measurements on the available, most standardized radiographs within the patients’ series, which may not be ideal. We do, however, believe that the noted evident radiographic toggling, with its consequences on the fracture reduction, was radiographically evident and can be attributed to the risk factors beyond the slight variations in the limb positioning.

## 5. Conclusions

Short proximal femoral nails may toggle in cases with wide medullary femoral canals if the lateral cortex is incompetent or the compression device does not fully engage the lateral cortex. The effect of femoral canal width on nail toggling and subsequent varus collapse may be abolished when the utilized lag device has an appropriate TAD and engages an intact lateral cortex. In patients with intertrochanteric fractures who have wide femoral canals, the utilization of long or intermediate cephalomedullary nails could be more reasonable whenever inadequate proximal fixation is anticipated.

## Figures and Tables

**Table 1:** Measurements’ results (Mean ± SD; (range)) for the non-complicated cases with good or acceptable reduction (80 cases).

Variable	Immediate Postop Tip Apex Distance	Last Follow-Up Tip Apex Distance	Immediate Postop Nail Shaft Angle	Last Follow-Up Nail Shaft Angle	Immediate. Postop Nail Tip to Medial Femoral Cortex Distance	Last Follow-Up Nail Tip to Medial femoral CORTEX Distance
**Mean ± SD** **(Median)**	16 ± 4.7 (15.9)	14.9 ± 4.8 (14.65)	−1.56 ± 2.21 (−1.35) (Negative value indicates a varus angle)	−2.56 ± 2.33 (−2.7)	6.92 ± 2.43 (6.85)	7.95 ± 2.67 (7.87)
**Wilcoxon signed-rank test**	***p* = 0.00014**		***p* ˂ 0.00001**		***p* < 0.00001**	

**Table 2:** The relationship between nail/canal ratio and TAD and nail toggling.

	Cases with a Toggle ≥ 4 Degrees and Varus Displacement	All Other Non-Complicated Cases, Excluding the Cases with Poor Reduction or AVN	Statistical Significance
**Number of cases**	16	80	
**Nail/canal ratio (Mean ± SD)**	0.593 ± 0.07	0.598 ± 0.08	*p* = 0.7 (Mann-Whitney U Test)
**Immediate postoperative TAD (Mean ± SD)**	15.7 ± 4.7	16 ± 4.7	*p* = 0.8 (Unpaired *t*-Test)

**Table 3:** Incidence of nail toggle ≥4 degrees in cases with lateral cortex incompetency, lag device non-engagement, or cases with combined factors (excluding five cases with poor surgical reduction or AVN) *. The incidence of complications was significantly higher in the cases with deficient proximal fixation.

	Cases with no Proximal Fixation Defect	Cases with Only the Lag Device Not Engaging the Lateral Cortex	Cases with Only Lateral Wall Incompetency	Cases with a Combination of the Two Factors
**Total Number of cases** (96) *	58	22	8	8
**Cases with nail toggle >4 degrees (%)**	0	2 (9.09%)	7 (87.5%)	7 (87.5%)
			Out of 16 cases with proximal lateral wall incompetency, 14 cases (87.5%) showed significant toggling.	
**Percent of complicated cases**	0/58 (0%)	16/38 (42.1%)		
**Fisher’s Exact test**	***p* < 0.0001 (Statistically significant)**			

**Table 4:** Incidence of deficient proximal fixation in complicated vs. uncomplicated cases (excluding the cases with poor surgical reduction or AVN). The incidence of proximal fixation defect was significantly higher in the cases with complications.

	Cases with Nail Toggling and Varus Displacement (16 Cases)	Cases Without Complications (80 Cases)
**Number of cases with deficient proximal fixation**	16 (100%)	22 (27.5%)
**Fisher’s Exact test**	***p* < 0.0001 (Statistically significant)**	

## Data Availability

The data presented in this study are available on request from the corresponding author after IRB approval and data use agreement.
